# Lack of Measles Transmission to Susceptible Contacts from a Health Care Worker with Probable Secondary Vaccine Failure — Maricopa County, Arizona, 2015

**DOI:** 10.15585/mmwr.mm6430a5

**Published:** 2015-08-07

**Authors:** Jefferson Jones, Ron Klein, Saskia Popescu, Karen Rose, Melissa Kretschmer, Alice Carrigan, Felicia Trembath, Lia Koski, Karen Zabel, Scott Ostdiek, Paula Rowell-Kinnard, Esther Munoz, Rebecca Sunenshine, Tammy Sylvester

**Affiliations:** 1 Epidemic Intelligence Service, CDC; 2 Maricopa County Department of Public Health, Phoenix, Arizona; 3 Phoenix Children’s Hospital, Arizona; 4 Health Systems Integration Program, CDC; 5 Career Epidemiology Field Officer Program, CDC

On January 23, 2015, the Maricopa County Department of Public Health (MCDPH) was notified of a suspected measles case in a nurse, a woman aged 48 years. On January 11, the nurse had contact with a patient with laboratory-confirmed measles associated with the Disneyland theme park–related outbreak in California (1). On January 21, she developed a fever (103°F [39.4°C]), on January 23 she experienced cough and coryza, and on January 24, she developed a rash. The patient was instructed to isolate herself at home. On January 26, serum, a nasopharyngeal swab, and a urine specimen were collected. The following day, measles infection was diagnosed by real time reverse transcription polymerase chain reaction testing of the nasopharyngeal swab and urine specimen and by detection of measles-specific immunoglobulin (Ig)M and IgG in serum by enzyme-linked immunosorbent assay. Because of her symptoms and laboratory results, the patient was considered to be infectious.

The case patient had documentation of receipt of 2 doses of measles-mumps-rubella (MMR) vaccine in 1991 and 1992. In 2006, the patient had received negative measles IgG serology test results; however, according to recommendations of the Advisory Committee on Immunization Practices, she was presumed to be immune because she had received two MMR doses (2).

The presence of measles IgG (index standard ratio = 8.2, with ≥1.1 considered seropositive) 2 days after rash onset suggested secondary vaccine failure (waning of vaccine-induced immunity, rather than failure to develop an immune response to administered vaccine [i.e., primary vaccine failure]). Symptoms in these patients range from typical measles to a much milder, modified illness (3). Secondary measles vaccine failure is uncommon, and although measles transmission from such persons has been documented (4), it is not believed to contribute significantly to spread (5).

The patient worked at a tertiary pediatric outpatient health care facility during January 20–21, a period which coincided with her infectious period. In cooperation with the health care facility, an investigation was conducted to prevent further transmission by identifying contacts, providing postexposure prophylaxis, recommending quarantine for unvaccinated contacts, and providing education for rapid isolation and diagnosis of symptomatic contacts (6). The health care facility identified 71 health care workers (HCWs) and 195 patients who had been exposed to the nurse on the 2 days she had worked; all 71 HCWs had documented receipt of ≥2 doses of MMR vaccine or serologic proof of measles immunity.

During January 26–30, the health care facility, in consultation with MCDPH, attempted to reach families of exposed patients by telephone; one to three telephone calls were made to each household. A total of 144 (74%) of 195 potentially exposed patients and their family members (total = 380 persons) were contacted (>72 hours after exposure). MMR vaccination status (receipt of ≥1 dose) and measles symptoms were ascertained by telephone interview for exposed patients and family members (Table). Fifty-one patients (among 47 families) could not be contacted, and the Arizona State Immunization Information System was accessed to verify their MMR vaccination status. The status of persons whose records listed no MMR vaccination history was considered unknown. Assuming that one adult (with unknown MMR vaccine status) accompanied each family, a total of 478 patients and family members were potentially exposed. Among the 478, 40 (8%) were considered to be potentially susceptible: 10 were unvaccinated persons without other evidence of measles immunity in non-high–risk groups (eight children aged 1–11 years and two adults aged 26 and 38 years), and 30 were persons in high-risk groups (21 infants aged <1 year, and therefore too young for routine MMR vaccination, and nine immunocompromised persons). Immune globulin was administered to 15 (71%) infants and eight (89%) immunocompromised patients within 6 days of their exposure.*

After 21 days had elapsed from the last measles exposure, calls to families of the 195 patients were attempted; 106 (54%) families responded and reported that no exposed family members had developed a febrile rash illness. No measles cases were reported in Maricopa County. These findings are consistent with previous reports demonstrating limited transmission from persons with secondary measles vaccine failure. In addition, the risk for transmission was reduced because all exposed HCWs had been vaccinated for measles.

HCWs born after 1956 should have documentation of receipt of 2 doses of MMR vaccine or laboratory evidence of measles immunity (2). Secondary vaccine failure occurs rarely, but transmission of measles to susceptible persons in these situations appears to be unlikely. If a patient is suspected of having measles, HCWs should implement airborne precautions (6). Case investigation and contact tracing should be conducted for all U.S. measles cases, regardless of vaccination history or occupation (6), and a history of travel should be solicited for any patient with a febrile rash illness (7). 2 doses of MMR vaccine, administered ≥28 days apart, are recommended for children aged ≥12 months and adults born after 1956, for prevention of measles.

## Figures and Tables

**TABLE:** Number of contacts* exposed to an MMR-vaccinated health care worker† with measles, by age group and MMR vaccination status — Maricopa County, Arizona, 2015

Age group	Total	Immunocompromised	History of measles disease	MMR vaccination status		

				≥1 dose	No doses	Unknown
0–11 months	21	0	0	0	21	0
1–17 years§	210	9	0	166	8	27
≥18 years§	228	0	2	145	2	79¶
Unknown	19	0	0	13	0	6
**Total**	**478**	**9**	**2**	**324**	**31**	**112**

**Abbreviation:** MMR = measles, mumps, and rubella.

* Includes only patients and their family members.

† Health care worker had documented receipt of two MMR doses, but history of negative measles IgG serology test results.

§ Includes 50 persons aged 1–17 years and one person aged ≥18 years using the Arizona State Immunization Information System (ASIIS) records for MMR history; any ASIIS records with no MMR vaccine history were considered unknown.

¶ Fifty-one patients (among 47 families) could not be contacted; assumed one adult accompanied each patient or family of patients for siblings (i.e., the parent or guardian).

## Footnotes

* Among seven persons who did not receive immune globulin, one had recently received an immune globulin dose, one could not be reached, one was traveling outside the country, two were contacted more than 6 days after exposure, and two refused.

## References

[1] Zipprich J, Winter K, Hacker J, Xia D, Watt J, Harriman K (2015). Measles outbreak—California, December 2014–February 2015. MMWR Morb Mortal Wkly Rep.

[2] McLean HQ, Fiebelkorn AP, Temte JL, Wallace GS (2013). Prevention of measles, rubella, congenital rubella syndrome, and mumps, 2013: summary recommendations of the Advisory Committee on Immunization Practices (ACIP). MMWR Recomm Rep.

[3] Lievano FA, Papania MJ, Helfand RF (2004). Lack of evidence of measles virus shedding in people with inapparent measles virus infections. J Infect Dis.

[4] Rota JS, Hickman CJ, Sowers SB, Rota PA, Mercader S, Bellini WJ (2011). Two case studies of modified measles in vaccinated physicians exposed to primary measles cases: high risk of infection but low risk of transmission. J Infect Dis.

[5] Rosen JB, Rota JS, Hickman CJ (2014). Outbreak of measles among persons with prior evidence of immunity, New York City, 2011. Clin Infect Dis.

[6] Kutty P, Rota J, Bellini W, Redd SB, Barskey A, Wallace G, Wharton M, Roush S (2008). Measles [Chapter 7]. Manual for the surveillance of vaccine-preventable diseases.

[7] Wilson ME (2014). Fever in Returned Travelers. CDC Health Information for International Travel 2014.

